# Gallic Acid Alleviates Hypertriglyceridemia and Fat Accumulation via Modulating Glycolysis and Lipolysis Pathways in Perirenal Adipose Tissues of Rats Fed a High-Fructose Diet

**DOI:** 10.3390/ijms19010254

**Published:** 2018-01-15

**Authors:** Da-Wei Huang, Wen-Chang Chang, Heng-Jui Yang, James Swi-Bea Wu, Szu-Chuan Shen

**Affiliations:** 1 Department of Food and Beverage Management, China University of Science and Technology, No. 245, Sec. 3, Academia Road, Taipei 11581, Taiwan; dawei0906@cc.cust.edu.tw; 2 Graduate Institute of Food Science and Technology, National Taiwan University, P.O. Box 23-14, Taipei 10672, Taiwan; d99641001@ntu.edu.tw (W.-C.C.); jsbwu@ntu.edu.tw (J.S.-B.W.); 3 Department of Human Development and Family Studies, National Taiwan Normal University, No. 162, Sec. 1, Heping East Road, Taipei 10610, Taiwan; 698060442@ntnu.edu.tw

**Keywords:** gallic acid, high-fructose diet, hypertriglyceridemia, insulin signaling, fat accumulation

## Abstract

This study investigated the ameliorative effect of gallic acid (GA) on hypertriglyceridemia and fat accumulation in perirenal adipose tissues of high-fructose diet (HFD)-induced diabetic rats. The previous results showed that orally administered GA (30 mg/kg body weight) for four weeks significantly reduced the levels of plasma glucose and triglyceride (TG) in HFD rats. GA also markedly decreased the perirenal adipose tissues weight of HFD rats in present study (*p* < 0.05). Western blot assay indicated that GA restored expression of insulin signaling-related proteins, such as insulin receptor (IR), protein kinase C-zeta (PKC-ζ), and glucose transporter-4 (GLUT4) in the perirenal adipose tissues of HFD rats. Moreover, GA enhanced expression of glycolysis-related proteins, such as phosphofructokinase (PFK) and pyruvate kinase (PK), and increased the expression of lipolysis-related proteins, such as adipose triglyceride lipase (ATGL), which is involved in lipolysis in the perirenal adipose tissues of HFD rats. This study revealed that GA may alleviate hypertriglyceridemia and fat accumulation through enhancing glycolysis and lipolysis pathways in perirenal adipose tissues of HFD rats. These findings also suggest the potential of GA in preventing the progression of diabetes mellitus (DM) complications.

## 1. Introduction

Diabetes mellitus (DM) is one of the fastest growing chronic diseases and is caused by a deficiency in insulin secretion or by ineffectiveness in insulin action [1]. Dysfunction of insulin requirements is involved in the derangement of carbohydrate, protein, and lipid metabolism, leading to numerous complications, such as hyperlipidemia, coronary artery disease, renal failure, neuropathy, retinopathy, and blindness [1,2]. More than 95% of diabetic patients have type 2 DM (T2DM), causing hyperglycemia via an ineffectiveness of insulin action or an inability to mount a normal response to insulin in peripheral cells, resulting in the symptoms of insulin resistance [3,4]. Fructose can cause hypertriglyceridemia, low density lipoprotein-cholesterol (LDL-C), weight gain, blood pressure elevation, and impaired glucose tolerance [5]. Long-term fructose intake may enhance oxidative stress, impair the antioxidant system, and increase lipid peroxidation [6,7]. It can also be associated with the pathogenesis of metabolic syndrome, including insulin resistance, abdominal obesity, dyslipidemia, intraabdominal fat accumulation, fatty liver, inflammation, and endothelial dysfunction, resulting in T2DM ultimately [5]. High-fructose diet (HFD)-induced diabetic rats are widely used as an in vivo model to investigate the mechanism of therapy for T2DM-associated insulin resistance [8]. 

Phenolic compounds are widely distributed in the plant kingdom. Plant-derived polyphenol compounds exhibit various pharmacological properties, which has been the subject of considerable interest in recent research [4]. Gallic acid (GA), an endogenous polyphenol in plants, is abundant in vegetables, grapes, berries, tea, fruit juices, and wine [1]. GA consists of one aromatic ring, three hydroxyl groups, and one carboxylic acid group. GA exhibits the strong antioxidant capacity due to the fact that three hydroxyl groups are linked to the aromatic ring in the ortho position. GA has been reported to exhibit pharmacological activities, including antioxidant, anti-obesity, anti-inflammatory, antimutagenic, and anticancer activity [9,10]. Moreover, GA exhibits antihyperglycemic, anti-lipid peroxidative, and antioxidant activities in streptozotocin (STZ)-induced diabetic rats [11]. In these rats, the oral treatment with GA resulted in a significant decrease in the levels of blood glucose, hepatic lipid peroxidation products, glycoprotein components, lipids and the activity of hydroxymethylglutaryl-CoA reductase, and a significant increase in levels of plasma insulin and liver glycogen [11].

An HFD-induced diabetic rat model has been reported to present the pathophysiological properties of T2DM in humans such as insulin resistance, glucose intolerance, dyslipidemia, renal impairment, and hypertension [12]. High fructose intake is linked to the prevalence of hyperglycemia, hypertriglyceridemia, obesity, and other metabolic syndromes [8]. Very few studies have examined the effect of GA on fat accumulation in adipose tissues of diabetes. The perirenal adipose tissue is the relatively large size in the intra-abdominal cavity and facilitates to cause a mass increase when compared with other adipose tissue [13]. The aim of the present study is to investigate the effect of GA on hypertriglyceridemia and fat accumulation in perirenal adipose tissues of HFD-induced diabetic rats. 

## 2. Results

### 2.1. Effect of GA on Weight of Perirenal and Epidydimal Adipose Tissues in HFD-Induced Diabetic Rats

**Figure 1:**
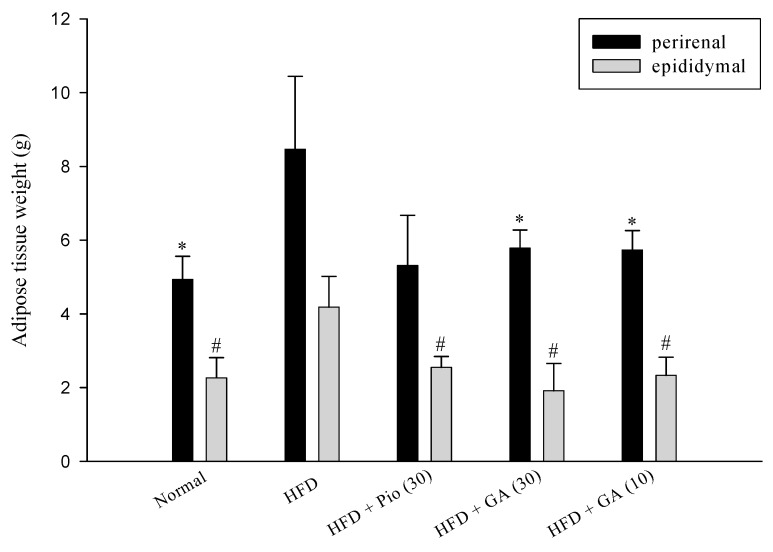
The perirenal and epididymal adipose tissues weight of HFD rats after being fed with GA for four weeks. GA: gallic acid; Normal: Normal rats were treated with saline; HFD: High fructose diet rats were treated with saline; HFD + Pio (30): High fructose diet rats treated with pioglitazone (30 mg/kg body weight); HFD + GA (30): High fructose diet rats treated with gallic acid (30 mg/kg body weight); HFD + GA (10): High fructose diet rats treated with gallic acid (10 mg/kg body weight). Values calculated as mean ± SD for six rats in each group. * *p* < 0.05, versus HFD rat for perirenal adipose. # *p* < 0.05, versus HFD rat for epididymal adipose tissue.

Perirenal and epididymal adipose tissue from rats was acquired and weighed after sacrifice. The results indicated that HFD increased perirenal and epidydimal adipose weight by 71.6% and 84.5% in comparison to the normal group, respectively. However, administration of 10 mg/kg body weight GA diminished the weight of perirenal and epidydimal adipose by 32.3% and 44.2% in HFD rats (*p* < 0.05), treatment of 30 mg/kg body weight GA caused 31.7%, and 54.1% decrease in HFD rats (*p* < 0.05) (Figure 1).

### 2.2. Effect of GA on Insulin Signal Transduction in the Perirenal Fat of HFD Rats

**Figure 2:**
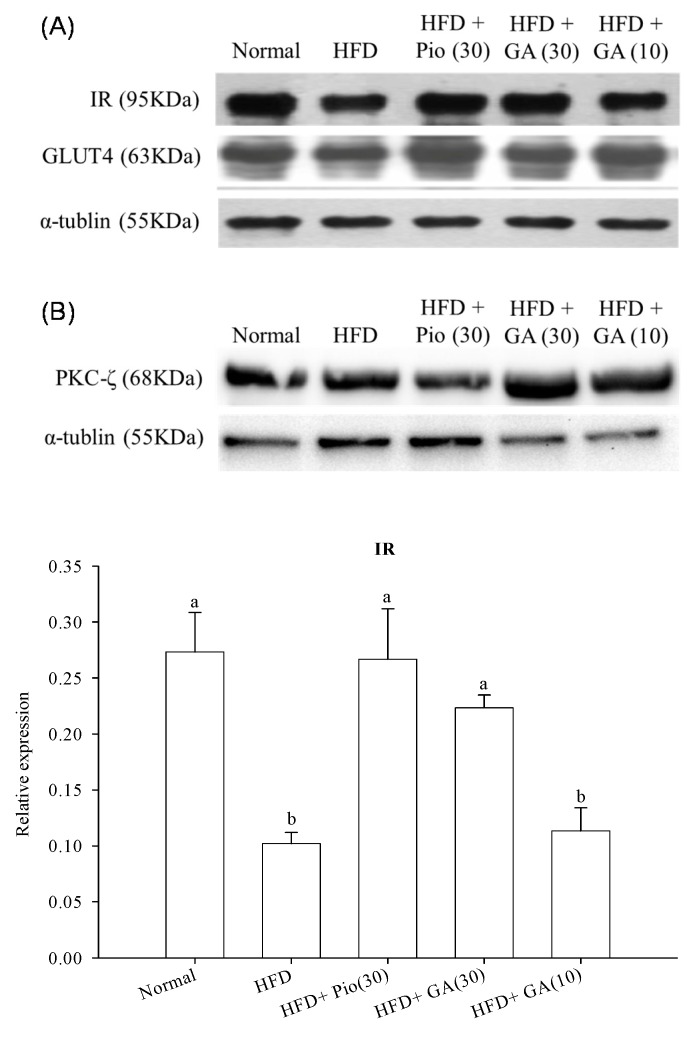

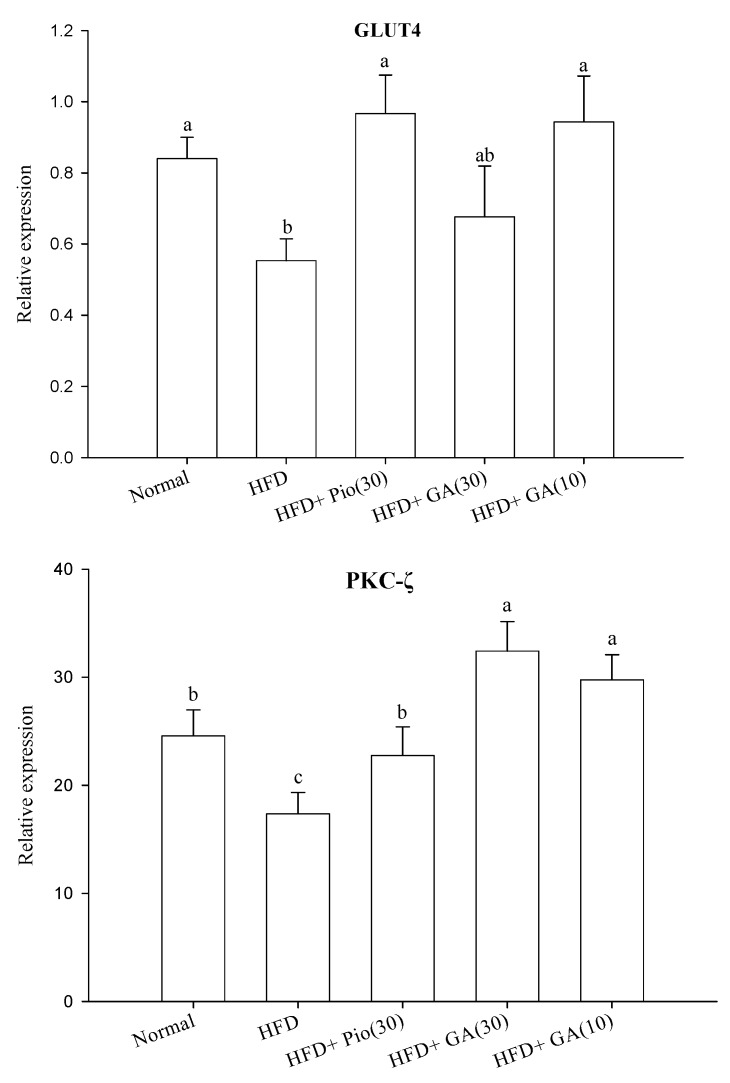
Effect of GA on the expression of insulin signal transduction-related proteins (**A**) insulin receptor (IR) and GLUT4, and (**B**) PKC-ζ in adipose tissues of HFD rats. GA: gallic acid; IR: insulin receptor; GLUT4: glucose transporter 4; PKC-ζ: protein kinase C-zeta; Normal: rats fed a normal diet; HFD: rats fed a 66% fructose diet; HFD + Pio (30): rats fed a 66% fructose diet and orally administered pioglitazone (30 mg/kg body weight); HFD + GA (30): rats fed a 66% fructose diet and orally administered GA (30 mg/kg body weight); HFD + GA (10): rats fed a 66% fructose diet and orally administered GA (10 mg/kg body weight). Different letters (a–c) indicate a significant difference at *p* < 0.05. Values calculated as the means ± SD for six rats in each group. The relative expressions of IR, GLUT4, and PKC-ζ in each treatment group were calculated using α-tublin as the standard.

Figure 2 shows that HFD significantly decreased the expression of IR by 67.1% in normal rats. Administration of 10 or 30 mg/kg body weight GA increased IR expression by 18.9% and 51.5% in HFD rats, respectively (*p* < 0.05) (Figure 2A). HFD also led to a 34.5% decrease in GLUT4 expression in the normal rats (*p* < 0.05) (Figure 2A). Administration of 10 or 30 mg/kg body weight GA remarkably restored the expression of GLUT4 by 70.5% and 22.3% when compared with the negative control group (*p* < 0.05) (Figure 2A). The expression of PKC-ζ declined by 29.3% in HFD rats compared with the normal group (*p* < 0.05) (Figure 2B). Administration of 10 or 30 mg/kg body weight GA enhanced PKC-ζ expression by 71.3% and 86.6%, respectively, in HFD rats (*p* < 0.05) (Figure 2B).

### 2.3. Effect of GA on Carbohydrate Metabolism and Lipid Metabolism in the Perirenal Fat of HFD Rats

**Figure 3:**
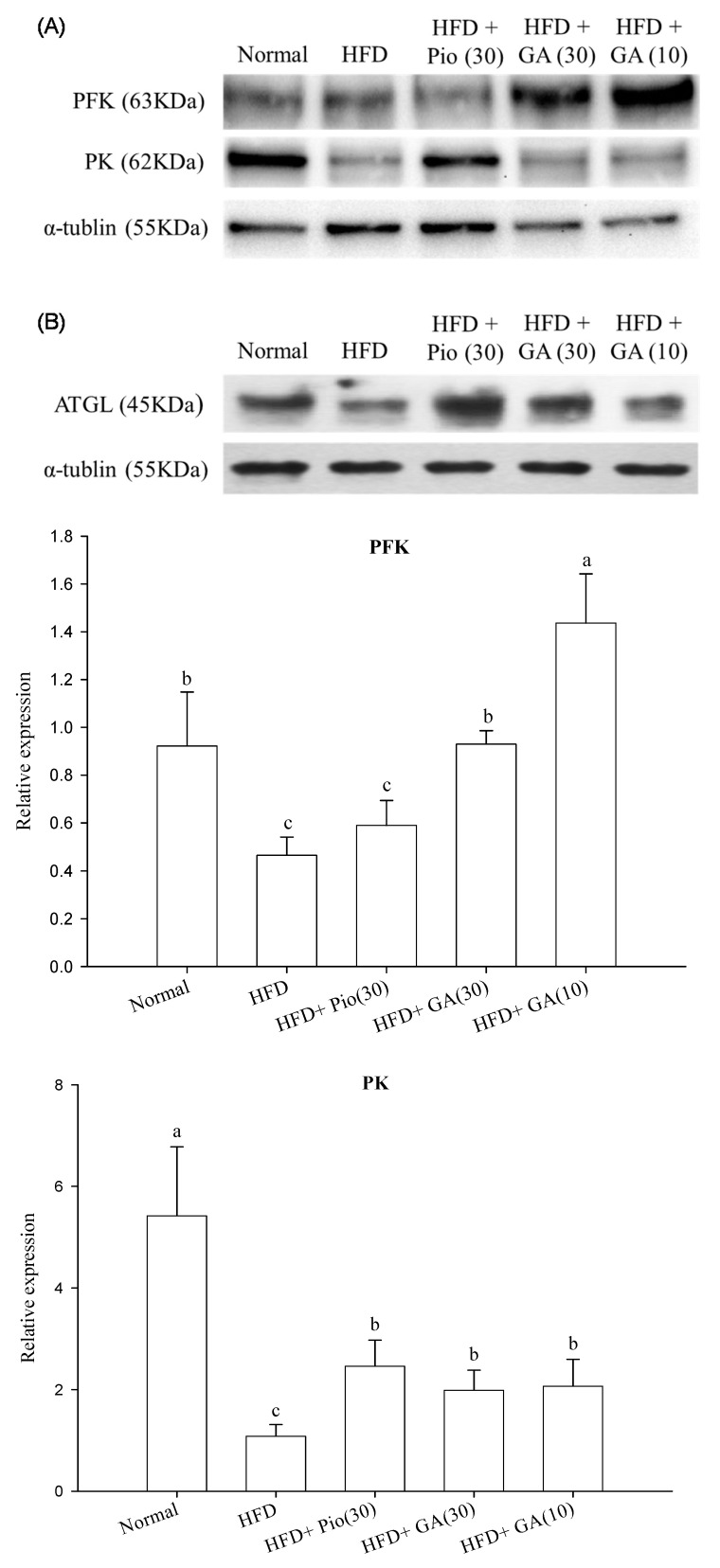

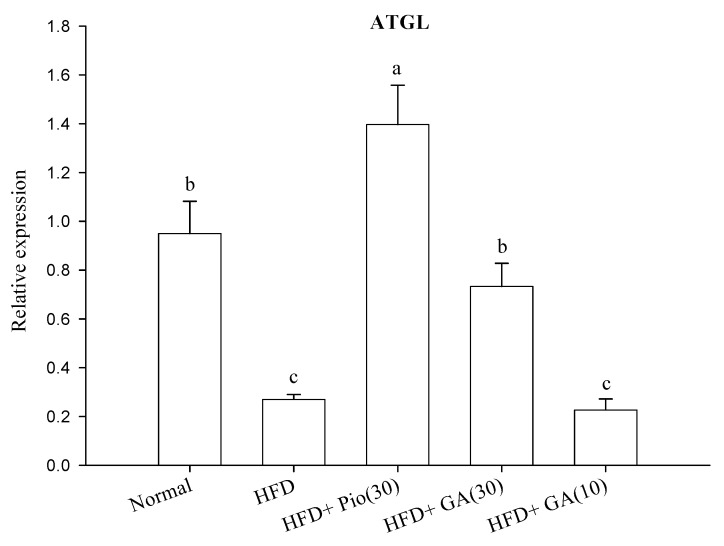
Effect of GA on the expression of carbohydrate (**A**) and lipid (**B**) metabolism-related proteins in adipose tissue of HFD rats. GA: gallic acid; PFK: phosphofructokinase; PK: pyruvate kinase; ATGL: adipose triglyceride lipase; Normal: rats fed a normal diet; HFD: rats fed a 66% fructose diet; HFD + Pio (30): rats fed a 66% fructose diet and orally administered pioglitazone (30 mg/kg body weight); HFD + GA (30): rats fed a 66% fructose diet and orally administered GA (30 mg/kg body weight); HFD + GA (10): rats fed a 66% fructose diet and orally administered GA (10 mg/kg body weight). Different letters (a–c) indicate a significant difference at *p* < 0.05. Values calculated as the means ± SD for six rats in each group. The relative expressions of PFK, PK, and ATGL in each treatment group were calculated using α-tublin as the standard.

HFD rats exhibited a 49.4% decrease in phosphofructokinase (PFK) expression as compared with the normal group (*p* < 0.05) (Figure 3A). Treatment with 10 or 30 mg/kg body weight GA significantly increased PFK expression by 208.3% and 99.6%, respectively, in HFD animals (*p* < 0.05) (Figure 3A). The expression of pyruvate kinase (PK) was suppressed by 80.0% in this group (*p* < 0.05) (Figure 3A). HFD rats administered 10 and 30 mg/kg body weight GA had restored PK expression, by 91.1% and 83.7%, respectively (*p* < 0.05) (Figure 3A). The rats fed with HFD also had a 71.6% decrease in expression of adipose triglyceride lipase (ATGL), which was enhanced by 171.6% in HFD rats treated with 30 mg/kg body weight GA (*p* < 0.05) (Figure 3B).

## 3. Discussion

The antioxidant propriety of GA is demonstrated to prevent the progression of diabetic complications [14]. GA is also reported to exhibit antihyperglycemic, anti-lipid peroxidative, and antioxidant effects on STZ-induced diabetic rats [11], and to ameliorate impaired glucose and lipid homeostasis in HFD-induced non-alcoholic fatty liver disease mice [9]. Our previous study indicated that GA ameliorates hyperglycemia and improves hepatic carbohydrate metabolism in HFD rats. The present study is to evaluate the effect of GA on amelioration of insulin signal transduction and lipid and glucose metabolism in the perirenal adipose tissues of HFD-induced diabetic rats.

HFD rats have been widely used as models for evaluating insulin resistance as well as diabetes [7,8]. High fructose intake may cause hyperglycemia, increase oxidative stress, and decrease insulin sensitivity, leading to insulin resistance in liver, skeletal muscle, and adipose tissues [15]. The previous study illustrated that GA significantly increased the ability of glucose uptake in hepatocytes and lead to alleviate hyperglycemia in HFD rats [7]. In this study, we proposed that enhancing the glucose uptake in adipocytes may be also associated with the advantage of GA on decreasing plasma glucose in HFD rats.

High fructose dietary may promote de novo lipogenesis and cause the accumulation of endogenous TGs, resulting in hyperlipidemia [15]. Phenolic acid was previously verified to improve abnormal lipid metabolism via reducing serum TG and LDL-C levels and enhancing HDL-C levels in diabetic rats [1]. The results from the current study indicate that GA supplementation may cause a significant reduction in serum TG levels in HFD rats. A previous study indicated that the administration of HFD causes an increase in intra-abdominal adipose mass in rats [13]. Hsu et al. illustrated that GA decreases epidydimal and perirenal fat weight in HFD-induced diabetic animals [16]. The present study revealed that the administration of GA might significantly decrease epidydimal and perirenal adipose weight in HFD rats, which is consistent with these previous findings. Treatment with GA was reported to improve lipid profiles by inhibiting lipogenesis in the adipose tissue of diabetic rats [17]. Okuno et al. verified that administration of troglitazone may decrease the concentration of triglyceride and free fatty acids in the blood via increasing number of small adipocytes in white adipose tissue, resulting in the amelioration of insulin resistance [18]. GA also induces apoptosis in 3T3-L1 pre-adipocytes, resulting in decreasing pre-adipocyte proliferation [19]. We speculate that administration of GA may reduce perirenal fat accumulation by suppressing adipocytes proliferation, leading to a decrease in perirenal adipose tissues weight and plasma triglyceride level in HFD rats.

In normal conditions, insulin binds to IR, triggers tyrosine phosphorylation of IR, and subsequently enhances the expression of IR substrate (IRS), phosphatidylinositol-3-kinase (PI3K), and Akt/protein kinase B (PKB), thus inducing translocation of GLUT to the cell membrane for promoting uptake of glucose in peripheral tissues [20]. GLUT4 is the predominant GLUT in adipocytes and responsible for glucose uptake via activation of the PI3K-Akt (PKB) or PI3K-protein kinase C (PKC) pathway [21]. GA was reported to cause translocation and activation of GLUT4 through boosting PI3K activity and phosphorylation of Akt in adipose tissue of high-fat diet fed-streptozotocin-induced rats [22]. Furthermore, the ζ isotype of protein kinase C (PKC-ζ) is a member of the atypical PKC subfamily, which has been widely recognized as a key regulator of critical intracellular signaling pathways and stimulated the action of GLUT4 [23,24]. GA is also reported to enhance the expression of GLUT4 via activating the PI3K-PKC pathway in 3T3-L1 adipocytes, resulting in an increased glucose uptake [24]. Previous studies have indicated that the expression of IR, insulin receptor substrate 1 (IRS-1), PI3K, and Akt is decreased in the muscle tissue of HFD-induced diabetic rats [25]. In the current study, administration of GA improved impaired protein expression of GLUT4 via increased protein expression of IR and PKC-ζ in perirenal fat, suggesting that GA may ameliorate insulin-signaling cascades through the PI3K-PKC pathway in HFD-induced diabetic rats. 

Glycolysis is the first step in glucose metabolism and use. Earlier studies demonstrated that the activity of glycolysis-related enzymes is diminished in T2DM rats [4]. PFK and PK play roles as the rate-limiting enzyme and the final step of glycolysis, respectively, and are mainly modulated by insulin [26,27]. Impaired expression of PFK may be improved by metformin in skeletal muscle, liver, and adipose tissues of STZ-induced diabetic mice [26]. PK activity and mRNA expression are decreased in adipose tissue of type 1 DM patients and in an insulin-deficient diabetic animal model [28]. Previous studies indicated that insulin ameliorates the activity and expression of hepatic PK, leading to ameliorated hyperglycemia in alloxan monohydrate-treated rats [29]. Here, we found that GA may increase expressions of PFK and PK via improving insulin sensitivity, resulting in improving glycolysis and subsequently ameliorating glucose metabolism in the perirenal adipose tissues of HFD diabetic rats. 

**Figure 4:**
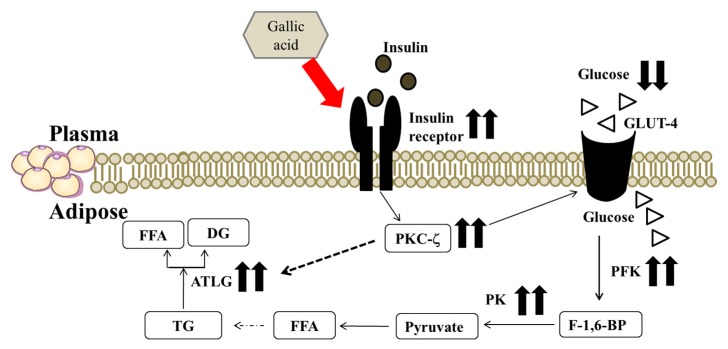
The postulated mechanism for GA to regulate carbohydrate and lipid metabolism via restoring insulin signaling, enhancing glycolysis and lipolysis pathways in perirenal adipocytes of high fructose diet-induced diabetic rats. GA: gallic acid; FFA: free fatty acid; DG: diglyceride; ATGL: adipose triglyceride lipase; TG: triglyceride; PKC-ζ: protein kinase C-zeta; PK: pyruvate kinase; GLUT-4: glucose transporter 4; PFK: phosphofructokinase; F-1,6-BP: fructose 1,6 biphosphate.

Adipocytes reduce lipolysis may cause maximizing triglyceride packaging into lipid droplets of adipocytes [30]. Ruegsegger et al. indicated that perirenal adipose tissue is the relatively large size in the intra-abdominal cavity [13]. In this study, we evaluated the ameliorative effect of GA on fat accumulation in perirenal adipose tissues in HFD rats. Dysfunctional lipolysis may influence energy homeostasis and the pathogenesis of obesity and insulin resistance [31]. Obesity is characterized by an increase in TG storage in adipose tissue [32]. ATGL is a key enzyme that is involved in intracellular degradation of triacylglycerol and is highly expressed in white and brown adipose tissue of mice and humans [33]. Earlier work showed that mRNA and protein expression of ATGL is reduced in the insulin-resistant state [32]. GA was reported to cause a significant decrease in triacylglycerol levels in HFD rats [7]. In the present study, the administration of 30 mg/kg body weight GA appeared to ameliorate the impaired ATGL expression in the perirenal fat of HFD diabetic rats. We speculated that GA may decrease the pathogenesis of obesity via promoting lipolysis in the HFD rat perirenal adipose tissues. We hypothesized that GA may ameliorate impaired insulin signal transduction and enhance glucose utilization via improving insulin sensitivity, and modulate lipid metabolism by the enhancement of ATGL expression, resulting in decreasing hyperglycemia and fat accumulation in perirenal adipose tissues of HFD rats as the consequence (Figure 4).

Oral administration of GA at a dose of 500 mg/kg was demonstrated to cause no toxicity or mortality in mice. GA is non-toxic at an acute dose of 5000 mg/kg body weight; this dose is recognized as no-observed-adverse-effect level (NOAEL) for GA in mice. A subacute administration of 1000 mg/kg body weight also confirmed its safety [34]. Subchronic toxicity of GA was investigated that the level of 119 and 128 mg/kg body weight/day were determined to be the NOAEL in male and female rats, respectively [35]. These levels of GA represent human equivalent doses of 19.4 and 20.48 mg in a 60 kg male and female in humans, respectively, as described by the FDA guidance for industry on conversion from rat to human equivalent doses [36]. Hence, we speculated that the administration of 30 mg/kg body weight GA (4.8 mg/kg body weight GA for human) has a high potential with the least toxicity in this study.

## 4. Materials and Methods

### 4.1. Chemicals

GA (purity > 97.5%, Analytical grade), Glucose Assay Kit, D-Glucose, fructose, pioglitazone hydrochloride, Tris-HCl, Triton X-100, and PMSF were purchased from Sigma (St. Louis, MO, USA). Anti-IR, anti-PKC-ζ, anti-GLUT4, anti-PFK, anti-PK, and anti-ATGL antibodies were purchased from Cell Signaling Technology (Beverly, MA, USA). All of the chemicals used in this study were of analytical grade.

### 4.2. Animals and Diets

Male Wistar rats (6 weeks old) were obtained from the National Laboratory Animal Center, Taipei, Taiwan. The room conditions and treatment procedures were in accordance with the National Institutes of Health Guide for the Care and Use of Laboratory Animal. All of the protocols were approved by the Institutional Animal Care and Use Committee of National Taiwan Normal University, Taipei, Taiwan (approval No. 103042, 23 December 2014). The rats were maintained under standard laboratory conditions of temperature 25 °C ± 1 °C and a 12-h light/12-h dark cycle with free access to food and water for the duration of the study. Wistar rats were fed a normal diet for two weeks and divided into five groups of six rats each. Group 1 was fed a normal diet for eight weeks. Group 2 was fed HFD throughout the experiment as a negative control. Group 3 received HFD for 8 weeks and was gavaged with pioglitazone (30 mg/kg) daily for the last four weeks as the positive control. Groups 4 and 5 were fed HFD (66% fructose in diet) for 8 weeks and gavaged with GA (10 or 30 mg/kg body weight, respectively) daily for the last four weeks of the eight-week period. The ingredients composition of experimental diets is according to our previous study [7].

### 4.3. Western Blot Analysis

The perirenal fat pad (1 g) was homogenized with 3 mL of RIPA buffer solution (ddw 8.7 mL/10× RIPA buffer 1 mL/100 mM PMSF 100 µL/Phosphatase Inhibitor Cocktail2 100 µL/Phosphatase Inhibitor Cocktail3 100 µL) at 4 °C for 10 min. The homogenate was centrifuged (3000 rpm, 5 min, 4 °C) to obtain the liquid at the bottom, which was centrifuged (15,000× *g*, 20 min, 4 °C) to acquire the cell protein for western blot analysis. 

Aliquots of the extract, each containing 40 μg of protein, were evaluated for the expression of IR, GLUT4, PKC-ζ, PFK, PK, and ATGL. The samples were subjected to 10% sodium dodecyl sulfate-polyacrylamide gel electrophoresis. The separated proteins were electrotransferred to a polyvinylidene difluoride membrane that was incubated with blocking buffer (phosphate-buffered saline containing 0.05% Tween-20 (PBST) and 5% *w*/*v* nonfat dry milk) for 1 h, washed three times with PBST, and then probed with 1:1000–1:2000 diluted solutions of anti-IR, anti-GLUT4, anti-PKC-ζ, anti-PFK, anti-PK, and anti-ATGL at 4 °C overnight. The intensity of the blots probed with 1:2000 diluted solutions of anti-α-tublin was used as a control to ensure that a constant amount of protein was loaded into each lane of the gel. The membrane was washed three times for 5 min each in PBST, and then incubated in a solution of horseradish peroxidase-conjugated anti-mouse IgG or anti-rabbit IgG secondary antibody, washed three times again for 5 min each in PBST, and exposed to the enhanced chemiluminescence reagent (Millipore, Darmstadt, Germany). Autoradiography was scanned and analyzed using a UVP Biospectrum image system (Level, Cambridge, UK).

### 4.4. Statistical Analysis

Data are presented as means ± SD after analysis using one-way ANOVA and Duncan’s new multiple-range tests with the Statistical Analysis System (SAS) version 9.3 (Cary, NC, USA). All of the comparisons were made relative to the controls or negative control. The differences were considered to be statistically significant at *p* < 0.01 or 0.05, indicated in tables and figures by distinct letters.

## 5. Conclusions

The present study demonstrates that administration of GA may be helpful in ameliorating carbohydrate and lipid metabolism abnormalities via restoring insulin signaling, increasing glycolysis and lipolysis-related protein expressions, which reduces fat accumulation in perirenal adipose tissues and alleviate hypertriglyceridemia in HFD-induced diabetic rats. Our findings support that GA exerts therapeutic effects and has the potential to be used in clinical medicine or as a dietary supplement on preventing the progression of complications in DM.

## Abbreviations

ATGL	Adipose triglyceride lipase
DG	Diglyceride
DM	Diabetes mellitus
F-1,6-BP	Fructose 1,6 biphosphate
FFA	Free fatty acid
GA	Gallic acid
GLUT4	Glucose transporter-4
HFD	High-fructose diet
IR	Insulin receptor
IRS	IR substrate
IRS-1	IR substrate 1
LDL-C	Low density lipoprotein-cholesterol
NOAEL	No-observed-adverse-effect level
PFK	Phosphofructokinase
PI3K	Phosphatidylinositol-3-kinase
PK	Pyruvate kinase
PKB	Akt/protein kinase B
PKC	Protein kinase C
PKC-ζ	Protein kinase C-zeta
STZ	Streptozotocin
T2DM	Type 2 DM
TG	Triglyceride
